# An Evaluation of the Effects of the Ruthenium (II)–Uracil Schiff Base Complex on Selected Markers of Glucose Homeostasis in Diet-Induced Prediabetic Male Rats

**DOI:** 10.3390/pharmaceutics18070811

**Published:** 2026-06-30

**Authors:** Zenele Chonco, Nompumelelo Anna-Cletta Gumede, Andile Khathi, Lindokuhle Mabuza-Mashaba

**Affiliations:** Department of Human Physiology, School of Medicine, College of Health Sciences, Westville Campus, University of KwaZulu-Natal, Durban 4000, South Africakhathia@ukzn.ac.za (A.K.);

**Keywords:** prediabetes, ruthenium complex, glucose homeostasis and dietary interventions

## Abstract

**Background**: The onset of type 2 diabetes mellitus (T2DM) is often preceded by prediabetes, a reversible state of insulin resistance and impaired glucose regulation driven by the chronic consumption of a high-calorie diet and sedentary lifestyle. Prediabetes is characterised by impaired glucose tolerance and elevated glycated haemoglobin (HbA1c). Although metformin improves insulin sensitivity, adherence limitations to lifestyle interventions highlight the need for alternative drugs that can be effective even without dietary intervention. In our laboratory, we synthesised a novel ruthenium complex that exhibits elevated biological activity. Accordingly, this study investigated the metabolic effects of a novel ruthenium (II)–uracil Schiff base complex in HFHC diet-induced prediabetic rats, with and without dietary intervention. **Methods**: Forty-eight male Sprague-Dawley rats (150–180 g) were divided into two groups, the standard diet (*n* = 12) and HFHC diet groups (*n* = 36), for prediabetic induction. Prediabetic animals were randomly assigned to respective treatment groups. The ruthenium complex was administered to prediabetic rats once a day, every third day, for 12 weeks, while monitoring changes in blood glucose, caloric intake, and body weight. **Results**: Diet-induced prediabetes resulted in increased fasting blood glucose and elevated HbA1c. The administration of the ruthenium (II)–uracil Schiff base complex reduced fasting blood glucose, improved insulin levels and ghrelin, enhanced GLUT4 expression, and significantly increased skeletal muscle glycogen, especially when combined with dietary intervention. **Conclusions**: The findings showed that the ruthenium complex exerts a pronounced effect on ameliorating glucose homeostasis, enhancing skeletal muscle glucose uptake, and improving overall metabolic function.

## 1. Introduction

Type 2 diabetes mellitus (T2DM) is a chronic metabolic disorder characterised by the abnormal metabolism of carbohydrates, fats, and proteins, contributing to chronic hyperglycaemia [1]. The onset of T2DM is often preceded by prediabetes [2,3]. Prediabetes is a metabolic condition where blood glucose levels are above the upper threshold for normal but below the threshold for a diagnosis of type 2 diabetes mellitus. Prediabetes is characterised by decreased insulin sensitivity, pancreatic β-cell dysfunction, and increased hepatic glucose production [4,5,6]. The prevalence of prediabetes was estimated to be 7.5% (374 million) in 2019 and is projected to reach 8.0% (454 million) by 2030 [7]. Prediabetes is primarily linked to the long-term consumption of unhealthy foods and a sedentary lifestyle, both of which are important risk factors for its development [8].

Mammalian target of rapamycin (mTOR) signalling pathways are involved in nutrient sensing and the control of food intake, which play a complex role in regulating the hormones ghrelin and leptin, which are crucial for appetite and energy balance [9,10]. mTOR integrates signals from these hormones and nutrient availability to influence food intake, energy expenditure, and the metabolism of fats and carbohydrates [11,12]. The upregulation of the mammalian target of rapamycin signalling pathway has been observed in prediabetes [13]. Another key element in the pathophysiology of prediabetes is glucose transporter 4 (GLUT4), which facilitates insulin-stimulated glucose uptake into skeletal muscle and adipose tissue [14].

Skeletal muscle plays a vital role in glucose clearance, accounting for over 80% of glucose uptake after an oral glucose load [15]. Insulin regulates this process by promoting the movement of vesicles containing GLUT4 to the plasma membrane, enabling efficient glucose absorption into muscle and adipose tissue [16,17]. When the relationship between insulin action and secretion is disrupted, the activity of insulin in tissues such as the liver and muscle, as well as insulin secretion, is affected, leading to insulin resistance and impaired glucose transporter 4 membrane translocation and resulting in hyperglycaemia [18,19]. Several studies have shown that prediabetes is a reversible condition that can be managed through a combination of pharmacological therapies and lifestyle interventions [20].

Metformin, a widely used biguanide derived from herbal sources, is the first-line treatment for type 2 diabetes mellitus [21]. It primarily functions by increasing insulin sensitivity in the liver, thereby reducing hepatic glucose production. In skeletal muscle, metformin enhances insulin action, leading to increased nonoxidative glucose disposal [22,23]. Although the long-term use of pharmacotherapy is effective and safe for managing prediabetes, low patient compliance with dietary modifications has been reported, which diminishes the overall effectiveness of metformin and increases the risk of developing type 2 diabetes mellitus [24,25,26]. Therefore, there is a need for novel drugs that remain effective even without dietary modifications.

Metal ions are increasingly recognised as regulators of glucose metabolism [27]. Biological processes depend on trace amounts of metals, and they require metal cofactors such as iron and zinc. Chromium and vanadium have been investigated as antidiabetic factors. Chromium, copper, zinc, and vanadium have been reported to exhibit insulin-mimetic properties [28,29,30]. Ruthenium compounds have demonstrated a range of pharmacological activities, with potential applications as anticancer, antibacterial, antioxidant, anti-inflammatory, and antidiabetic agents that can improve glycaemic control and insulin sensitivity [31,32,33,34]. The literature reports that ruthenium complexes exhibit low toxicity, however, among the investigated ruthenium complexes, biological activity was limited, primarily due to the low reactivity of the side groups and their oxidation states [35,36]. In our previous work, different ruthenium compounds underwent physicochemical characterisation and biological evaluation, and no acute toxicity or overt adverse effects were observed [37,38].

A novel uracil-based Schiff base compound, coordinated with ruthenium (II), was synthesised in our laboratory and has shown enhanced biological activity. The newly synthesised complexes were fully characterised, and their effects on glucose metabolism were evaluated in vitro using liver cells [39]. Although these in vitro results are promising, further in vivo studies are necessary to elucidate the precise mechanisms underlying the observed anti-hyperglycaemic effects. Animal models have significantly advanced diabetes research by allowing researchers to control genetic and environmental factors that influence disease development and its complications [40,41]. In this study, a diet-induced prediabetic rat model was used. This model successfully replicates key features of prediabetes, including insulin resistance, hyperglycaemia, and glucose intolerance. It closely reflects the clinical characteristics of the disease, and it is a valuable tool for investigating the causes of prediabetes and developing innovative therapeutic strategies for its management [42,43]. Therefore, using this animal model, this study sought to investigate the effect of a novel ruthenium (II) complex on glucose homeostasis in diet-induced prediabetic male Sprague-Dawley rats, in both the presence and absence of dietary intervention.

## 2. Methods and Materials

### 2.1. Chemicals and Drugs

Chemicals and drugs were sourced as follows: Metformin 500 mg/kg (Sigma-Aldrich, St. Louis, MO, USA). Dimethyl sulphoxide (DMSO), anthrone (Sigma-Aldrich, St. Louis, MO, USA). Ethanol, sodium sulphate (NaSO_4_), potassium hydroxide (KOH), sulphuric acid H_2_SO_4_ (Merck chemicals, Johannesburg, South Africa). Isofor, liquid nitrogen (Safeline Pharmaceuticals (Pty) Ltd., Roodeport, South Africa). High-fat high-carbohydrate (HFHC) diet (AVI Products (Pty) Ltd., Waterfall, South Africa) and ruthenium (II) complex, *trans*-[RuCl2(PPh3)(urdp)] 15 mg/kg (School of Chemistry and Physics, University of KwaZulu-Natal, Pietermaritzburg, South Africa).

### 2.2. Drug Preparation and Dosing

**Figure 1:**
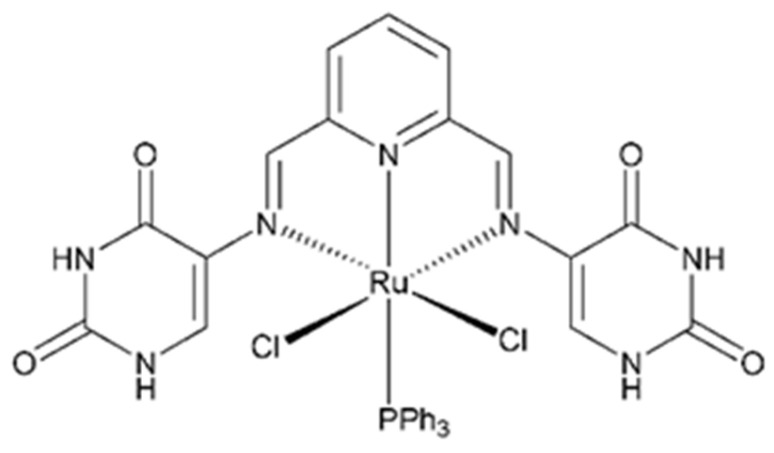
The molecular structure of the ruthenium (II) complex trans-[RuCl_2_(PPh_3_) (urdp)] [39].

Metformin tablets were finely crushed and suspended in DMSO immediately prior to administration. Metformin was administered orally at the specified dose of 500 mg/kg every third day throughout the treatment period. The *trans*-[RuCl2(PPh3)(urdp)] was prepared by dissolving the required amount in dimethyl sulphoxide and administered at a dose of 15 mg/kg. Dimethyl sulphoxide was used solely as a vehicle and administered at a fixed volume of 3 mL/kg body weight. All doses were adjusted according to body weight and administered under identical experimental conditions. The *trans*-[RuCl2(PPh3) (urdp)] used in this study was synthesised and characterised in previous work [39]. The molecular structure of the ruthenium (II) complex trans-[RuCl_2_(PPh_3_) (urdp)] investigated in this study is shown in Figure 1.

### 2.3. Animals and Housing

Male Sprague-Dawley rats (*n* = 48), 150–180 g, bred and housed in the Biomedical Research Unit (BRU) of the University of KwaZulu-Natal, were used in this study. These animals were housed in a room with a 12 h light/12 h dark cycle, at room temperature (25 °C), at relative humidity = 55 ± 5%, and with noise levels of less than 65 decibels for the duration of this study. The animals in each group had access to food and water ad libitum. All animal procedures and conditions were implemented in accordance with the University of KwaZulu-Natal Animal Research Ethics Committee (AREC/00006098/2023).

#### 2.3.1. Induction of Prediabetes

Animals were allowed to acclimatise to their new environment for a week while consuming standard rat chow before the commencement of the experimental diet. Experimental prediabetes was induced in these rats following a previously established protocol [40]. The animals were housed in groups of six in a type 4 large cage with a well-enriched environment. The bedding was changed every second day. After acclimatisation for a week, the animals were randomly assigned to the following diet groups: standard diet with drinking water (ND) (*n* = 12) and high-fat high-carbohydrate (HFHC) (*n* = 36) diet with both drinking water and 15% fructose (AVI Products (Pty) Ltd., Waterfall, South Africa) dissolved in water for 20 weeks. Prediabetes was induced by allowing the animals to feed on the HFHC diet for 20 weeks [28]. Glucose tolerance was evaluated 5 days after 20 weeks of induction using an oral glucose tolerance test to determine prediabetes according to ADA criteria. Animals with fasting blood glucose greater than 5.6 mmol/L were considered prediabetic and were further grouped for pharmacological studies. Animals that were fed the standard diet were also tested.

#### 2.3.2. Experimental Design

The animals were randomly divided into 8 groups of 6 animals each (12 normal, 36 prediabetic). In this study, there were 8 groups:

Group 1: Vehicle (DMSO 3 mL/kg) received a standard diet (ND); Group 2: Non-prediabetic control (NPD) received a standard diet (ND); Group 3: Prediabetic (PD) rats received a HFHC diet without treatment; Group 4: PD received a standard diet; Group 5: Metformin (MTF) group received an HFHC diet and was treated with an oral dose of metformin (500 mg/kg, Sigma-Aldrich, St. Louis, MO, USA); Group 6: MTF group received a standard diet and was treated with an oral dose of metformin (500 mg/kg, Sigma-Aldrich, St. Louis, MO, USA); Group 7: Ruthenium (RU) group received an HFHC diet and was treated with a ruthenium (II)–uracil Schiff base complex (15 mg/kg); Group 8: Received a standard diet and was treated with a ruthenium (II)–uracil Schiff base complex (15 mg/kg). The treatment period lasted 12 weeks, and animals were treated once a day every third day at 09:00 a.m.

In each group, monitored parameters such as FBG, blood triglycerides, food intake, fluid intake, and urine output were measured once after 4 weeks, while body weight was recorded weekly at the same time over 12 weeks. The animals were individually placed on metabolic cages (Makrolon polycarbonate metabolic cages (Techniplast, Labotec, Midrand, South Africa)) overnight (24 h) to monitor the parameters once after 4 weeks, then returned to colony cages.

#### 2.3.3. Blood Collection and Tissue Harvesting

After 12 weeks of treatment, all animals were anaesthetised with Isofor (100 mg/kg) (Safeline Pharmaceuticals (Pty) Ltd., Roodeport, South Africa) in a gas anaesthetic chamber (Biomedical Resource Unit, UKZN, Durban, South Africa) for 3 min. Blood was collected by cardiac puncture and injected into individual pre-cooled heparinised containers. The blood was then centrifuged (Eppendorf centrifuge 5403, Hamburg, Germany) at 4 °C, 503 g for 15 min. Plasma was collected and stored at −80 °C in a Bio Ultra freezer (Snijers Scientific, Tilburg, The Netherlands) until ready for biochemical analysis. Skeletal muscle and the liver were removed, rinsed with cold normal saline, and snap-frozen in liquid nitrogen before storage in a BioUltra freezer (Snijers Scientific, Tilburg, The Netherlands) at −80 °C until biochemical analysis.

### 2.4. Oral Glucose Tolerance (OGT) Response

To assess the glucose tolerance response of animals subjected to a modern HFHC diet over 20 weeks, an oral glucose tolerance test (OGTT) was performed following carbohydrate loading. The OGTT responses of all animal groups were monitored using our established laboratory protocol. Briefly, after a 12 h fasting period, glucose was measured (at time zero) before administering a monosaccharide syrup (glucose; 0.86 g/kg, p.o.) (RIIG) via oral gavage with an 18-gauge needle that is 38 mm long and curved and has a 21/4 mm ball end (Able Scientific, Canning Vale, Australia). Blood glucose levels were determined using the tail-prick method. Blood samples were collected and analysed with a OneTouch Select glucometer (Lifescan, Mosta, Malta, UK). Blood glucose concentrations were recorded at 15, 30, 60, and 120 min following carbohydrate administration.

### 2.5. Biochemical Analysis

The concentrations of glycated haemoglobin (HbA1c), ghrelin, and insulin were analysed in plasma using ELISA kits (Elabscience and Biotechnology, Wuhan, China) according to the manufacturer’s instructions. The homeostatic model assessment (HOMA), which is used to quantify insulin resistance and beta-cell function, was performed using the HOMA calculator. Glycogen analysis was conducted on the gastrocnemius muscle tissues. The assay followed a well-established laboratory protocol [42]. Gastrocnemius muscle tissues (50 mg) were weighed and heated with KOH (30%, 2 mL) at 100 °C for 30 min. Then, Na_2_SO_4_ (10%, 0.194 mL) was added to stop the reaction, and the mixture was allowed to cool. For glycogen precipitation, 200 µL of the cooled mixture was aspirated and mixed with 200 µL of ethanol (95%). The precipitated glycogen was pelleted, washed, and redissolved in water (1 mL). Subsequently, anthrone (0.5 g, dissolved in 250 mL of sulphuric acid (4 mL) was added, and the sample was boiled for 10 min. After cooling, absorbance was measured at 620 nm using the Spectrostar Nano spectrophotometer (BMG Labtech, Ortenburg, Baden-Württemberg, Germany). Glycogen concentrations were determined from a standard curve.

### 2.6. Quantitative Real-Time PCR

Ribonucleic acid (RNA) was extracted from skeletal tissue samples collected during week 12 of the treatment period. RNA extraction was performed using the Maxwell^®^ RSC simplyRNA Tissue Kit (Promega, Madison, WI, USA). RNA yield was measured with a NanoDrop, and RNA concentration was standardised (Thermo Fisher, Waltham, MA, USA). Complementary deoxyribonucleic acid (cDNA) was then synthesised using the cDNA synthesis kit. SYBR Green, I master mix was utilised for amplification according to the manufacturer’s instructions on the LightCycler system (Bio-Rad Laboratories, Sandton, South Africa). The cycling conditions commenced with pre-incubation at 95 °C for 60 s, followed by a 3-step amplification over 45 cycles at 95 °C for 15 s, 60 °C for 30 s, and 72 °C for 30 s. Melting curve analysis was conducted at 95 °C for 10 s, 65 °C for 60 s, and 97 °C for 1 s. Cooling was achieved at 37 °C for 30 s. Glyceraldehyde-phosphate dehydrogenase (GAPDH) served as an internal control to normalise the data and determine the relative expression of the target gene. Gene expression levels were calculated using the 2^−ΔΔCt^ method. Glucose transporter 4 gene expression was analysed using the following primers: forward primer, 5′ AGGCCGGGACACTATACCC 3′, reverse primer, 5′ ACTTTCTGTGGGGCGTTGAT 3′.

### 2.7. Statistical Analysis

All data were expressed as the mean ± standard error of mean (SEM). Statistical analysis was performed using GraphPad Prism Software (version 8.0.2, GraphPad Software, San Diego, CA, USA), with a one-way analysis of variance (ANOVA) followed by Tukey–Kramer multiple-comparison test. Values of *p* < 0.05 indicate statistical significance between the groups.

## 3. Results

### 3.1. Effects of Ruthenium (II)–Uracil Schiff Base Complex on Fasting Blood Glucose and Plasma Insulin

Fasting blood glucose (FBG) and plasma insulin were measured in all experimental groups at the end of the treatment period (week 12). There was a significant increase in *FBG* in the prediabetic group (PD) compared to the non-prediabetic group (NPD) (*p* < 0.05). The groups that received dietary intervention showed significantly decreased FBG levels compared to the prediabetic group (*p* < 0.05). Notably, there was a significant difference in plasma insulin concentration between the PD, HFHC + MET, and HFHC + RU groups compared with the NPD group (*p* < 0.05), as shown in Table 1. Using FBG and plasma insulin levels, the HOMA2-IR index for all animals was calculated as the product of plasma glucose and insulin at the end of the treatment period. The results showed that the HOMA2-IR index differed significantly among all experimental groups compared with the NPD group (*p* < 0.05). However, the combination of dietary intervention and ruthenium significantly decreased the HOMA2-IR index compared with the prediabetic group, as shown in Table 1.

### 3.2. Oral Glucose Tolerance Test Response

**Figure 2:**
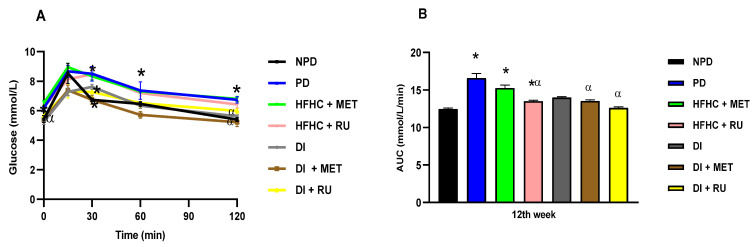
Effect of ruthenium (II)–uracil Schiff base complex on OGTT response in prediabetic rats with or without dietary intervention. Values are expressed as mean ± SEM (*n* = 6). * *p* < 0.05 in comparison to non-prediabetic (NPD) control. α *p* < 0.05 in comparison to prediabetic (PD) control. Metformin and high-fat high-carbohydrate (HFHC + MET); ruthenium (II)–uracil Schiff base complex and high-fat high-carbohydrate (HFHC + RU); diet intervention (DI). Metformin and normal diet (DI + MET); normal diet and ruthenium (II)–uracil Schiff base complex (DI + RU). (**A**) Oral glucose tolerance test (OGTT) showing fasting blood glucose concentrations measured at 0, 30, 60, 90, and 120 min following glucose loading at week 12. (**B**) Area under the curve (AUC) calculated from the OGTT data for all experimental groups.

The OGTT and area under the curve (AUC) were measured at the end of the treatment period (12th week) in all the groups. The oral glucose tolerance test (OGTT), Figure 2A, and the corresponding area under the curve (AUC), Figure 2B, are shown. Compared with the NPD group, the PD control group showed a significantly higher FBG concentration at time 0, before glucose loading (*p* < 0.05). Following glucose loading, the FBG concentration of PD remained higher at all time intervals during the OGTT as compared to the NPD group, as shown in Figure 2A. Interestingly, the groups treated with the ruthenium (II)–uracil Schiff base complex had fasting glucose concentrations within the range of those in the NPD group (*p* < 0.05).

### 3.3. Glycated Haemoglobin Concentration (HbA1c)

**Figure 3:**
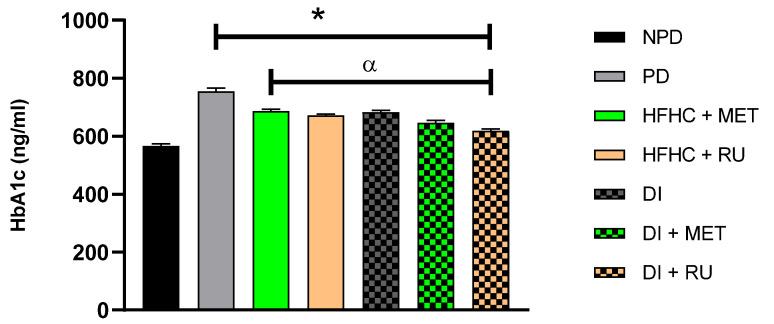
Effect of ruthenium (II)–uracil Schiff base complex on HbA1c concentration in prediabetic rats with or without dietary intervention. Values are expressed as mean ± SEM (*n* = 6). * *p* < 0.05 in comparison to non-prediabetic (NPD) control. α *p* < 0.05 in comparison to prediabetic (PD) control. Metformin and high-fat high-carbohydrate (HFHC + MET); ruthenium (II)–uracil Schiff base complex and high-fat high-carbohydrate (HFHC + RU); diet intervention (DI). Metformin and normal diet (DI + MET); normal diet and ruthenium (II)–uracil Schiff base complex (DI + RU).

At the end of the treatment period, all the experimental groups were analysed for HbA1c concentration (Figure 3). The HbA1c concentration in all experimental groups was significantly higher than that in the NPD group (*p* < 0.05). However, the HbA1c concentrations of those treated groups with or without diet intervention, HFHC + MET, HFHC + RU, DI, DI + MET, and HFHC + RU decreased significantly in comparison to PD (*p* < 0.05).

### 3.4. Caloric Intake

**Figure 4:**
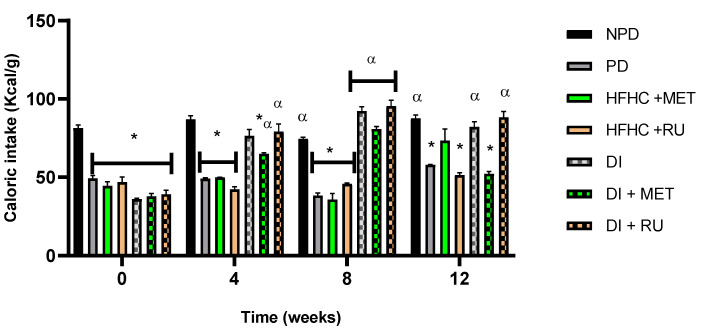
Effect of ruthenium (II)–uracil Schiff base complex on caloric intake in prediabetic rats with or without dietary intervention. Values are presented as mean ± SEM (*n* = 6). * *p* < 0.05 compared to non-prediabetic (NPD) control. α *p* < 0.05 compared to prediabetic (PD) control. Metformin and high-fat high-carbohydrate (HFHC + MET); ruthenium (II)–uracil Schiff base complex and high-fat high-carbohydrate (HFHC + RU); diet intervention (DI). Metformin and normal diet (DI + MET); normal diet and ruthenium (II)–uracil Schiff base complex (DI + RU).

The caloric intake of all experimental groups was measured every four weeks from the start of the treatment period (week 0 to week 12) (Figure 4). At week 0, all experimental groups had lower caloric intake than the non-prediabetic (NPD) group. By week 4, the dietary intervention groups showed a significant increase in food intake (*p* < 0.05). Additionally, the administration of the ruthenium (II)–uracil Schiff base complex resulted in a significant increase in caloric intake at week 8 compared with the PD group (*p* < 0.05), as shown in Figure 4.

### 3.5. Effects of Ruthenium (II)–Uracil Schiff Base Complex on Body Weight

**Figure 5:**
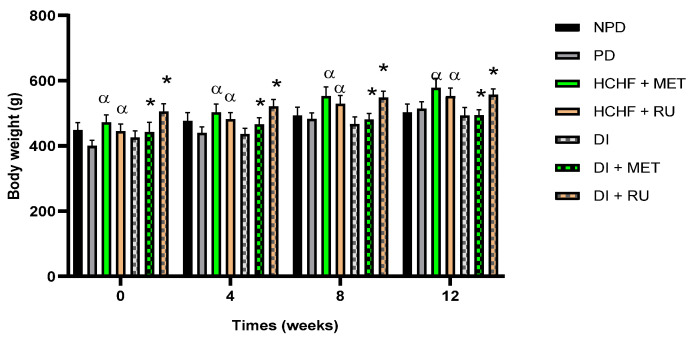
Effects of ruthenium (II)–uracil Schiff base complex on body weight in prediabetic rats with or without dietary intervention. Values are presented as mean ± SEM (*n* = 6). * *p* < 0.05 compared to non-prediabetic (NPD) control. α *p* < 0.05 compared to prediabetic (PD) control. Metformin and high-fat high-carbohydrate (HFHC + MET); ruthenium (II)–uracil Schiff base complex and high-fat high-carbohydrate (HFHC + RU); diet intervention (DI). Metformin and normal diet (DI + MET); normal diet and ruthenium (II)–uracil Schiff base complex (DI + RU).

The body weights of the animals were monitored throughout the experiment, as shown in Figure 5. The combination of dietary intervention and treatment groups (DI + MET and DI + RU) led to a significant difference in body weight compared with the non-prediabetic (NPD) group (*p* < 0.05). The HFHC + MET group and HFHC + RU group showed significant body weight gain compared with the prediabetic group (PD) (*p* < 0.05), as shown in Figure 5.

### 3.6. Ghrelin Concentrations

**Figure 6:**
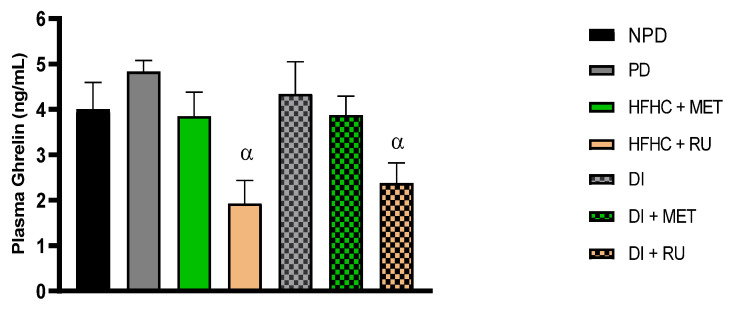
Effect of ruthenium (II)–uracil Schiff base complex on ghrelin concentration in prediabetic rats with or without dietary intervention. Values are expressed as mean ± SEM (*n* = 6). α *p* < 0.05 in comparison to prediabetic (PD) control. Metformin and high-fat high-carbohydrate (HFHC + MET); ruthenium (II)–uracil Schiff base complex and high-fat high-carbohydrate (HFHC+ RU); diet intervention (DI). Metformin and normal diet (DI + MET); normal diet and ruthenium (II)–uracil Schiff base complex (DI + RU).

The plasma concentration of ghrelin was measured in all the experimental groups at the end of the treatment period. There was no significant difference between the NPD and PD groups; however, the ruthenium (II)–uracil Schiff base complex-treated groups showed a significant decrease compared with the PD group (*p* < 0.05), as shown in Figure 6.

### 3.7. Skeletal Muscle Glycogen Concentration

**Figure 7:**
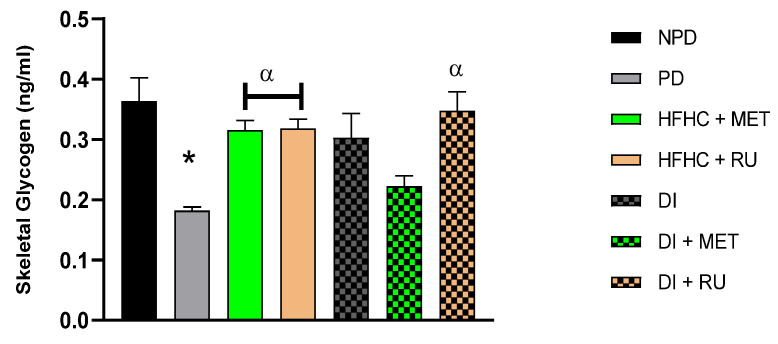
Effect of ruthenium (II)–uracil Schiff base complex on skeletal muscle glycogen in rats with or without diet intervention. Values are expressed as mean ± SEM (*n* = 6). * *p* < 0.05 in comparison to non-prediabetic (NPD) control. α *p* < 0.05 in comparison to prediabetic (PD) control. Metformin and high-fat high-carbohydrate (HFHC + MET); ruthenium (II)–uracil Schiff base complex and high-fat high-carbohydrate (HFHC+ RU); diet intervention (DI). Metformin and normal diet (DI +MET); normal diet and ruthenium (II)–uracil Schiff base complex (DI + RU).

Skeletal muscle glycogen concentrations were measured at the end of the treatment period. The results showed that skeletal muscle glycogen concentrations in the PD group were significantly lower than in the NPD group. Moreover, the skeletal muscle glycogen in the HFHC + MET, HFHC + RU and DI + RU groups was significantly increased when compared to the PD group (*p* < 0.05), as shown in Figure 7.

### 3.8. Skeletal Muscle GLUT 4 Expression

**Figure 8:**
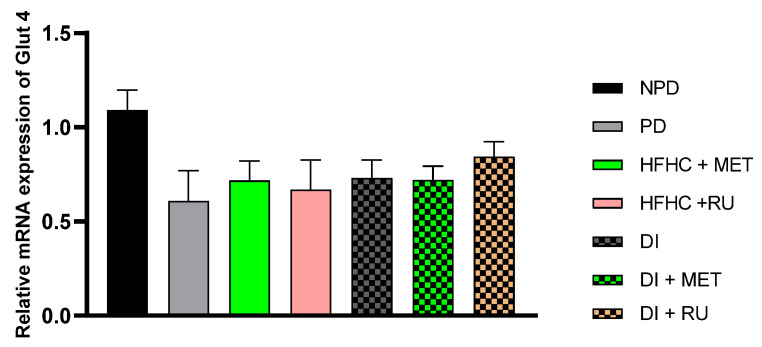
Effects of ruthenium (II)–uracil Schiff base complex on GLUT (glucose transporter) 4 expression in rats with or without diet intervention after treatment period of 12 weeks. Values are expressed as mean ± SEM (*n* = 6). Metformin and high-fat high-carbohydrate (HFHC + MET); ruthenium (II)–uracil Schiff base complex and high-fat high-carbohydrate (HFHC+ RU); diet intervention (DI). Metformin and normal diet (DI + MET); normal diet and ruthenium (II)–uracil Schiff base complex (DI + RU).

GLUT4 expression was quantified in skeletal muscle collected at the end of week 12. A decrease was observed in all experimental groups compared to the NPD group; however, the difference was not statistically significant. Both the ruthenium (II)–uracil Schiff base complex and metformin treatments had similar effects, increasing expression compared to the PD group. No significant differences were found among all groups, as shown in Figure 8.

## 4. Discussion

The onset of type 2 diabetes mellitus is a condition characterised by impaired insulin action and secretion, often preceded by a condition known as prediabetes [5,36]. Prediabetes is a reversible metabolic state marked by moderate insulin resistance, β-cell dysfunction, and chronic hyperglycaemia [37,38]. Prediabetes, mainly caused by the chronic consumption of unhealthy diets and sedentary lifestyles, involves disruptions in pathways such as the mTOR pathway and impaired GLUT4-mediated glucose uptake in skeletal muscle, the primary site of postprandial glucose clearance [9,39]. While metformin effectively improves insulin sensitivity, its success is limited by poor adherence to lifestyle changes, emphasising the need for therapies that work independently of dietary interventions [35,36].

Ruthenium-based compounds have emerged as promising agents with various biological activities, including potential antidiabetic effects [32,33,34]. A novel ruthenium (II)–uracil Schiff base complex synthesised in our laboratory has demonstrated encouraging in vitro effects on glucose metabolism [39]. However, further in vivo studies are necessary to clarify the mechanisms behind the glucose-lowering effects. Therefore, the current study aimed to investigate the impact of a novel ruthenium (II) complex on glucose homeostasis in diet-induced prediabetic male Sprague-Dawley rats, with and without dietary intervention.

Glucose homeostasis is the regulated balance between glucose production, glucose uptake, and glucose utilisation that maintains blood glucose concentrations within physiological range [38,44,45,46,47,48]. This regulation ensures a steady energy supply, especially for the brain, and relies on coordinated hormonal signals and the actions of key organs, such as the liver and skeletal muscle. When this regulatory balance is disrupted, it compromises insulin signalling and glucose clearance, increasing the risk of metabolic disorders such as prediabetes and type 2 diabetes mellitus [49,50,51,52]. Prediabetes is an early, reversible state in which insulin signalling, glucose uptake, and metabolic regulation begin to decline. It is characterised by impaired fasting glucose, impaired glucose tolerance, or elevated glycated haemoglobin A1c [38,53,54,55].

The combination of DI + RU showed further improvement than the ruthenium complex alone, suggesting a complementary interaction between dietary modification and treatment and providing protection against progression to overt type 2 diabetes mellitus. Recent studies on a ruthenium complex show that it has hypoglycaemic effects on glucose homeostasis [20,56,57,58]. The results of the present study confirm those of a study by Makanyane et al. (2024) investigating the hypoglycaemic effects of the same ruthenium complex in cells [39]. Nutrition is an important factor in promoting and maintaining good health. Consuming healthy, nutrient-balanced foods, such as fruit, vegetables, and protein, has been reported to provide significant protection against the development of disorders [59,60,61,62].

Under normal conditions, eating an HFHC diet delivers an excessive nutrient load to the body, requiring metabolic organs to manage increased levels of glucose, fatty acids, and triglycerides [63,64,65,66,67]. The long-term consumption of an HFHC diet has been associated with the disruption of normal energy balance and lipid accumulation in metabolically active organs, leading to inflammation, oxidative stress, and impaired metabolic signalling [68,69,70,71,72]. The consistent consumption of an HFHC diet has also been linked to significant increases in body weight, visceral adiposity, and dyslipidaemia, due to its higher calorie content compared to a standard healthy diet and the development of prediabetes [73,74,75].

The findings of the present study indicated that the prediabetic group showed reduced caloric intake and lower body weight compared with the non-prediabetic group, indicating that the HFHC diet provides high energy density, allowing animals to meet their metabolic needs with less food while still driving metabolic overload. This is confirmed by the significant increase in FBG and the decreased insulin sensitivity observed in the prediabetic group. The dietary intervention alone in rats significantly increased caloric intake, resulting in increased body weight gain compared to the prediabetic group. The metformin-treated group showed a significant increase in body weight compared with the PD group, in both the presence and absence of dietary intervention.

The administration of the ruthenium (II)–uracil Schiff base complex significantly increased body weight compared with the prediabetic group over time, with significant effects in the HFHC diet group. When compared with NPD, the HFHC + RU group led to more weight gain, whereas the DI + RU group had a similar weight gain to the NPD group, but their weight often remained higher over time. Ruthenium complex treatment consistently produced higher weight gain efficiency under both dietary conditions. Compared with metformin, the ruthenium complex resulted in higher body weight in both dietary conditions, indicating greater weight gain efficiency. Previous studies revealed that metformin prevents weight gain or promotes mild weight loss by activating AMPK, enhancing peripheral insulin sensitivity, and reducing appetite [42,76,77,78]. The findings of the present study confirm that the ruthenium (II)–uracil Schiff base complex exerts a metabolic effect that extends beyond body weight control. This was also evident in the way the ruthenium complex decreased FBG and enhanced insulin sensitivity, promoting nutrient storage or reducing energy expenditure, thereby improving glucose homeostasis.

Ghrelin is a hormone that stimulates appetite and influences hunger regulation [79,80,81]. It is the only known circulating hormone that targets both peripheral and central areas to increase food intake and promote fat storage [82,83]. In healthy individuals, plasma ghrelin levels fall after eating and gradually rise before the next meal. Circulating plasma ghrelin levels are linked with the regulation of food intake and energy balance [84,85]. Insulin plays a key role in lowering post-meal ghrelin levels. In prediabetic individuals, the usual postprandial decline in ghrelin is impaired, indicating an abnormal reduction in circulating ghrelin after meals [86,87,88]. The results of this study showed that the prediabetic group had higher plasma ghrelin levels than the non-prediabetic group, suggesting a disruption in ghrelin regulation before or after meals. Dietary intervention alone lowered ghrelin levels to prediabetic levels, indicating some improvement. The metformin-treated groups showed a non-significant decrease in plasma ghrelin compared with the prediabetic group and showed ghrelin effects similar to those of the non-prediabetic group.

The administration of the ruthenium (II) complex, in both the absence and presence of a dietary intervention, led to a significant decrease compared with the prediabetic group. The ruthenium complex suppresses ghrelin more effectively than metformin, suggesting the normalisation of gut–brain–pancreas signalling that complements peripheral insulin sensitisation [89,90]. Despite lower ghrelin levels, the ruthenium complex, especially without dietary intervention, caused significant increases in body weight and late-phase caloric intake compared to prediabetic conditions, indicating a ghrelin-independent mechanism that promotes weight gain, likely through diet-related improvements in energy storage efficiency [91]. The ruthenium complex may stimulate lipogenesis or insulin signalling or decrease energy expenditure. Compared with a non-prediabetic control, the ruthenium complex suppresses ghrelin but leads to more body weight gain, again emphasising ghrelin-independent pathways, such as dietary composition and altered energy expenditure [92,93,94].

The ruthenium (II) complex suppressed plasma ghrelin levels more effectively than metformin, indicating the normalisation of appetite-regulating alongside improved insulin sensitivity. Despite this ghrelin suppression, the ruthenium complex still promotes weight gain, suggesting that its metabolic actions are mediated by enhanced nutrient storage and energy expenditure. Glycogen is the main form of glucose storage in the liver and skeletal muscles. It serves as a readily accessible source of glucose for energy production [49,95]. Under normal physiological conditions, high plasma glucose levels trigger insulin secretion, which activates glycogen synthase and promotes glycogen formation in both the liver and muscles [96,97]. In the prediabetic state, skeletal muscle glycogen levels decrease due to impaired glucose uptake from insulin resistance. When glycogen stores are abundant, insulin action, glucose transporter 4 translocation, and muscle glucose uptake are sufficient; when levels are low, insulin deficiency or resistance occurs.

Indeed, in this study, skeletal muscle glycogen was significantly lower in the prediabetic group than in the NPD group, indicating impaired insulin-mediated glucose disposal and/or reduced glycogen synthase activity [17]. In the dietary intervention alone group, glycogen concentration increased, suggesting improved glucose homeostasis. The HFHC + MET group showed a significant increase in glycogen content, and the DI + MET group showed a non-significant increase, compared with the prediabetic group. Treatment with the ruthenium complex, in both the presence and absence of dietary conditions, resulted in a significant increase in glycogen concentration compared with the prediabetic group. The level of skeletal muscle glycogen in the DI + RU group almost normalises towards that in the NPD group.

The ruthenium complex had a significant effect on glycogen concentrations compared with the prediabetic group, indicating the restoration of glycogen storage capacity. In comparison, metformin produced only a moderate improvement, whereas the ruthenium complex nearly returned glycogen levels to those seen in normal physiological conditions. The effects of the ruthenium complex on weight might be independent of ghrelin; the glycogen results indicate improved muscle glucose handling, especially with dietary intervention. The ruthenium complex significantly increased glycogen levels above those of the prediabetic group, both in the absence and in the presence of dietary interventions. These findings suggest that the ruthenium (II) complex enhances glucose homeostasis by improving insulin sensitivity, thereby increasing skeletal muscle glycogen storage.

Glucose transporter 4 is the main insulin-responsive glucose transporter found in skeletal muscle and adipose tissue, where it plays a critical role in post-meal glucose clearance [17,88]. When insulin activates the PI3K–AKT signalling pathway, glucose transporter 4 -containing vesicles translocate to the cell membrane, increasing cellular glucose uptake [15,88,89]. The level of glucose transporter 4 mRNA reflects the muscle’s capacity to produce the transporter and respond effectively to insulin. In healthy metabolic conditions, higher glucose transporter 4 expression enhances insulin-stimulated glucose uptake, supports glycogen synthesis, and helps maintain overall glucose homeostasis [15,90,91].

Chronic insulin resistance, lipid accumulation, and inflammation suppress glucose transporter 4 gene transcription, lowering the number of available transporters and further weakening the muscle’s ability to clear glucose [92,93,94]. The pathophysiology of the glucose transporter 4 pathway reflects a combination of signalling defects, transcriptional downregulation, and impaired vesicle transportation, all of which disrupt normal glucose homeostasis [95,96]. The present study’s findings showed a non-significant decrease in glucose transporter 4 gene expression in the prediabetic group compared with the NPD group, suggesting a disturbance in normal glucose homeostasis. The dietary intervention alone increases GLUT4 gene expression compared with the prediabetic group. The metformin-treated groups showed an increased effect on GLUT4 gene expression compared with the prediabetic group, indicating improved glucose handling by skeletal muscle. The ruthenium (II)–uracil Schiff base complex, in both the presence and absence of dietary conditions, increased glucose transporter 4 gene expression compared with the prediabetic group, with the DI + RU group having almost normalised its GLUT4 gene expression towards that of the NPD group. Both metformin and ruthenium complex treatments increased glucose transporter 4 gene expression compared with the prediabetic group; however, there were no statistically significant differences among the groups.

The ruthenium complex partially restored glucose transporter 4 gene expression compared with the prediabetic group, with the effect being more pronounced under dietary interventions. The ruthenium complex alone did not fully normalise glucose transporter 4 to NPD levels. Under DI, the ruthenium complex showed more upregulation than metformin, mirroring the glycogen results. The ruthenium complex improves muscle glucose handling, leading to better glycogen storage when dietary quality is supportive. The present study findings suggest that ruthenium complexes support muscle glucose transporter 4 transcription and glycogen, especially with DI, and promote weight gain not driven by ghrelin, suggesting more energy storage efficiency. This suggests that the ruthenium complex enhances glucose homeostasis by restoring glucose transporter 4 expression and increasing skeletal muscle glycogen via the amelioration of insulin sensitivity. These findings indicate that ruthenium promotes muscle glucose handling and metabolic efficiency, positioning it as a promising therapeutic agent for improving early defects in prediabetic glucose regulation [97,98].

The biological effects observed in the present study can be meaningfully compared with those reported by Mabuza et al. (2018) [36]. The two complexes differ in their coordination environments, ligand denticity, and overall charge distribution and demonstrate glucose-lowering and insulin-sensitising effects in experimental models. The present results indicate that trans-[RuCl_2_(PPh_3_) (urdp)] likewise exerts favourable effects on glucose metabolism, extending these observations to a prediabetic animal model. Together, these findings suggest that subtle variations in ligand architecture within this family of ruthenium (II) complexes may modulate biological activity while preserving antidiabetic potential.

## 5. Conclusions

The findings of this study demonstrated that diet-induced prediabetes disrupts multiple aspects of metabolic regulation, including glucose control, appetite signalling, muscle glucose uptake, and energy storage. Dietary intervention improved fasting glucose, insulin sensitivity, ghrelin regulation, and muscle glycogen, highlighting the reversibility of early metabolic dysfunction. The administration of metformin produced moderate improvements across these parameters, consistent with its known effects on hepatic glucose output and insulin action. In contrast, the ruthenium (II)–uracil Schiff base complex exerted broader metabolic benefits: it lowered fasting glucose, suppressed ghrelin, enhanced glucose transporter 4 expression, and significantly increased skeletal muscle glycogen, especially when combined with dietary intervention. Despite reducing the primary appetite-stimulating hormone, ruthenium increased body weight and caloric efficiency, indicating a ghrelin-independent shift toward enhanced nutrient storage. While prompting the need for further investigations, these findings suggest that the ruthenium complex improves glucose homeostasis and muscle glucose handling more effectively than metformin, offering a promising multi-target strategy for preventing progression from prediabetes to type 2 diabetes mellitus.

## Figures and Tables

**Table 1:** Effects of ruthenium (II)–uracil Schiff base complex on fasting blood glucose, fasting plasma insulin, and HOMA-IR values in prediabetic animals during 12-week treatment period, with or without dietary intervention. Values are shown as means ± SEM (*n* = 6). * *p* < 0.05 compared to non-prediabetic (NPD) control. α *p* < 0.05 compared to prediabetic (PD) control. Metformin and high-fat high-carbohydrate (HFHC + MET); ruthenium (II)–uracil Schiff base complex and high-fat high-carbohydrate (HFHC + RU); diet intervention (DI). Metformin and normal diet (DI + MET). Ruthenium (II)–uracil Schiff base complex and normal diet (DI + RU).

Groups	Fasting Blood Glucose (mmol/L)	Plasma Insulin (mIU/L)	HOMA-IR Values
NPD	5.3 ± 0.2	1.0 ± 0.1	1.4 ± 0.2
PD	6.5 ± 0.1 *	0.8 ± 0.2 *	6.2 ± 0.3 *
HFHC + MET	5.8 ± 0.2	1.3 ± 0.2 *	4.6 ± 0.3 *
HFHC + RU	5.8 ± 0.1	1.3 ± 0.2 *	5.2 ± 0.2 *
DI	5.1 ± 0.2 α	1.2 ± 0.2 *	4.9 ± 0.2 *
DI + MET	5.4 ± 0.08 α	1.0 ± 0.2 α	3.9 ± 0.3 *
DI + RU	5.3 ± 0.09 α	1.2 ± 0.1 α	4.0 ± 0.2 *α

## Data Availability

The raw data supporting the conclusions of this article will be made available by the authors, without undue reservation.

## Abbreviations

The following abbreviations are used in the manuscript:

ADA	American Diabetes Association
AMPK	AMP-activated protein kinase
ANOVA	One-way analysis of variance
AUC	Area under the curve
BRU	Biomedical research unit
cDNA	Complementary deoxyribonucleic acid
DI	Diet intervention
DMSO	Dimethyl sulphoxide
FBG	Fasting blood glucose
GAPDH	Glyceraldehyde-phosphate dehydrogenase
GLUT4	Glucose transporter 4
HbA1c	Glycated haemoglobin
HFHC	High-fat high-carbohydrate
HOMA	Homeostatic model assessment
KOH	Potassium hydroxide
MET	Metformin
mTOR	Mammalian target of rapamycin
NPD	Non-prediabetic group
OGTT	Oral glucose tolerance test
PCR	Polymerase chain reaction
PD	Prediabetic group
PI3K–AKT	Phosphoinositide 3-kinase/protein kinase B
RNA	Ribonucleic acid
RU	Ruthenium complex
T2DM	Type 2 diabetes mellitus
UKZN	University of KwaZulu-Natal
